# Biventricular vortex ring formation corresponds to regions of highest intraventricular viscous energy loss in a Fontan patient: analysis by 4D Flow MRI

**DOI:** 10.1007/s10554-017-1250-8

**Published:** 2017-09-30

**Authors:** Vivian P. Kamphuis, Arno A. W. Roest, Jos J. M. Westenberg, Mohammed S. M. Elbaz

**Affiliations:** 1 Department of Pediatrics, Division of Pediatric Cardiology, Leiden University Medical Center, Leiden, The Netherlands; 2 Netherlands Heart Institute, Utrecht, The Netherlands; 3 Department of Radiology, Leiden University Medical Center, Leiden, The Netherlands

## Image focus

**Fig. 1:**
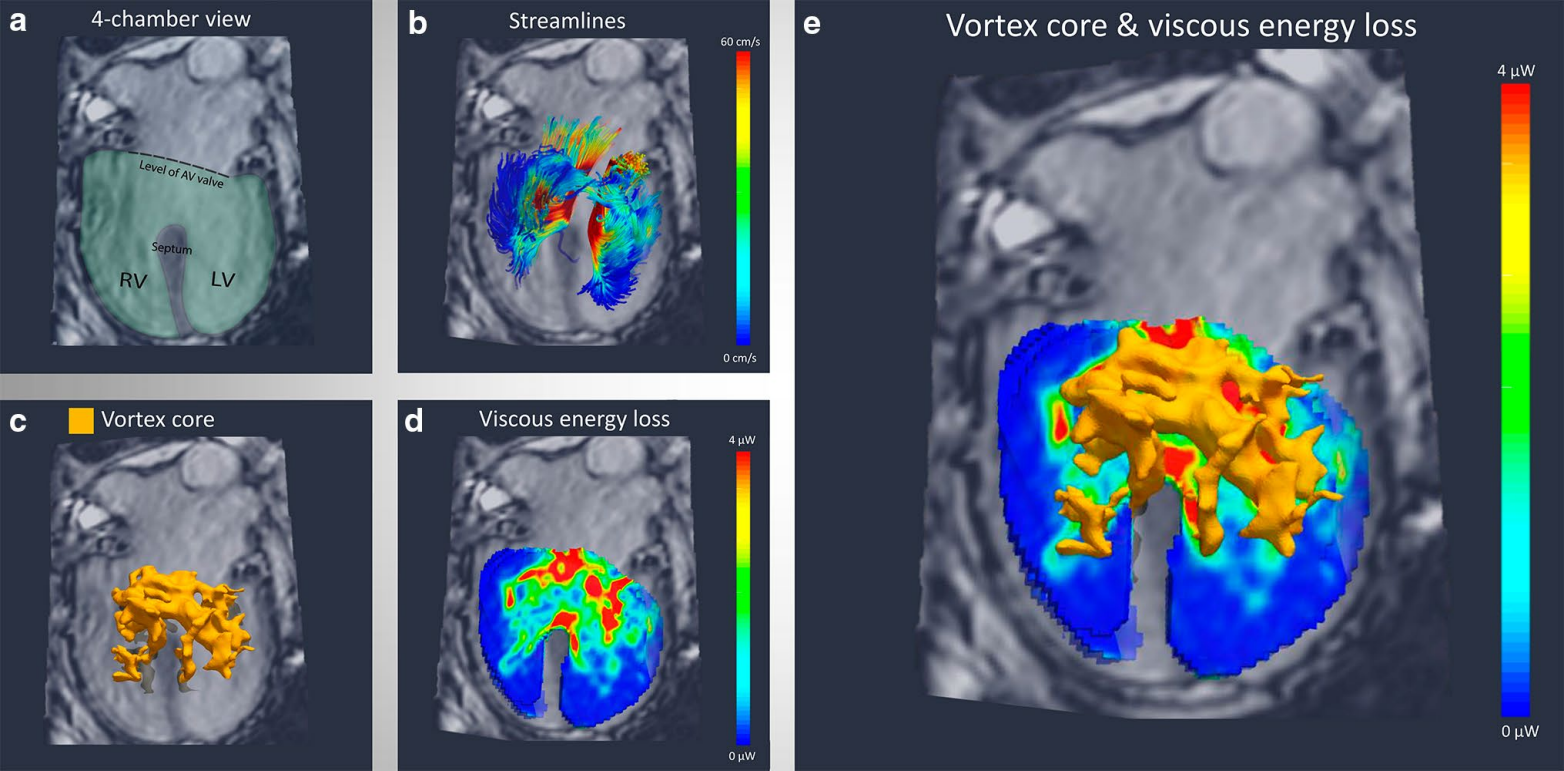
Vortical flow and viscous energy loss in a patient with a Fontan circulation

An adult patient with a Fontan circulation because of a complete atrioventricular septal defect (Fig. 1a) and a double outlet right ventricle with pulmonary stenosis, underwent a magnetic resonance imaging (MRI) scan including whole-heart four-dimensional (4D) Flow MRI as part of routine follow-up. 4D Flow MRI was used to assess intracardiac vortical flow patterns and in vivo viscous energy loss (EL)-the kinetic energy that is irreversibly lost due to viscosity-induced frictional forces-during diastole around peak early filling [1].

4D Flow MRI-derived streamline visualization showed a complex three-dimensional (3D) inflow pattern from the common atrioventricular valve with the formation of vortical flow around the ventricular part of the septal defect (Fig. 1b). Mathematical identification of vortex core structures from 4D Flow MRI [2] showed a large 3D ring-like vortex formation filling the common biventricular region at the level of the ventricular septal defect, with protrusion in both ventricles (Fig. 1c). To assess the regions of highest EL in this patient, a map of EL inside the ventricle was made in the cross-sectional four-chamber view (Fig. 1d). In Fig. 1e, regions of the identified 3D vortex structure associate with the highest EL levels in the EL map. These high EL levels anatomically correspond to the region of the ventricular septal defect and part of the remaining septum.

Knowledge on the consequences of intracardiac deformations on hemodynamic vortex formation and viscous energy loss might be useful in unraveling the pathophysiological mechanisms leading to circulatory failure, one of the main causes of morbidity and mortality in Fontan patients.
